# Cerebral Hemodynamic Changes to Transcranial Doppler in Asymptomatic Patients with Fabry’s Disease

**DOI:** 10.3390/brainsci10080546

**Published:** 2020-08-12

**Authors:** Carla Vagli, Francesco Fisicaro, Luisa Vinciguerra, Valentina Puglisi, Margherita Stefania Rodolico, Antonello Giordano, Raffaele Ferri, Giuseppe Lanza, Rita Bella

**Affiliations:** 1 Department of Medical and Surgical Sciences and Advanced Technologies, University of Catania, Via Santa Sofia 78, 95123 Catania, Italy; carlavagli@gmail.com; 2 Department of Biomedical and Biotechnological Science, University of Catania, Via Santa Sofia 89, 95123 Catania, Italy; drfrancescofisicaro@gmail.com; 3 Department of Neurology, Azienda Socio-Sanitaria Territoriale (ASST) Cremona, Viale Concordia 1, 26100 Cremona, Italy; luisa.vinciguerra@asst-cremona.it (L.V.); valentina.puglisi@asst-cremona.it (V.P.); 4 C.N.R. Institute for Biomedical Research and Innovation–IRIB, Section of Catania, Via P. Gaifami 18, 95126 Catania, Italy; margheritastefania.rodolico@cnr.it; 5 Department of Neurology, Guzzardi Hospital, Via Papa Giovanni XXIII, 97019 Vittoria, Italy; antonellomaria.giordano@gmail.com; 6 Department of Neurology IC, Oasi Research Institute-IRCCS, Via Conte Ruggero 73, 94018 Troina, Italy; rferri@oasi.en.it (R.F.); giuseppe.lanza1@unict.it (G.L.); 7 Department of Surgery and Medical-Surgical Specialties, University of Catania, Via Santa Sofia 78, 95123 Catania, Italy

**Keywords:** Fabry’s disease, cerebrovascular disease, white matter lesions, transcranial Doppler, cerebral blood flow, cerebral hemodynamics

## Abstract

Background: Patients with Fabry’s disease (FD) may be asymptomatic or show a spectrum of clinical manifestations, including cerebrovascular disease, mainly affecting posterior circulation. Few and conflicting studies on cerebral blood flow (CBF) velocity by transcranial Doppler sonography (TCD) in asymptomatic FD (aFD) subjects have been published. Our study aims to assess TCD in aFD subjects to identify any preclinical CBF change. Methods: A total of 30 aFD subjects were consecutively recruited and compared to 28 healthy controls. Brain magnetic resonance imaging was normal in all participants. TCD was used to study blood flow velocity and indices of resistance of intracranial arteries from the middle cerebral artery (MCA), bilaterally, and from the basilar artery (BA). Cerebral vasomotor reactivity (CVR) was also evaluated from MCA. Results: No difference was found between groups for MCA parameters of CBF velocity and CVR. Compared to controls, a higher mean blood flow velocity and a lower resistance index from BA were observed in FD subjects. No correlation was found between any BA-derived TCD parameter and the level of lyso-globotriaosylceramide. Conclusions: aFD subjects show evidence of altered CBF velocity in posterior circulation. Preclinical detection of neurovascular involvement in FD might allow appropriate management and prevention of future cerebrovascular complications and disability.

## 1. Introduction

### 1.1. Fabry’s Disease

Fabry’s disease (FD) is a rare, X-linked, inherited disorder of glycosphingolipid metabolism due to the lack or deficiency of lysosomal α-galactosidase A (α-galA) [1]. The *GLA* gene codes for the α-galA enzyme, which catalyzes the cleavage of the terminal galactose from globotriaosylceramide (Gb3) [2]. Absent or deficient activity results in a progressive Gb3 accumulation [3], and the hydrophilic deacylated derivative globotriaosylsphingosine (lysoGb3) is thought to have cytotoxic, proinflammatory, and profibrotic effects [4]. The accumulation is prominent in vascular endothelium, vascular smooth muscle cells, and pericytes [5,6], thus leading to vascular stenosis and occlusion.

FD may present with a wide spectrum of clinical manifestations, ranging from a severe phenotype in males, who have low or nonfunctional α-GalA activity, to asymptomatic disease in some females. Typically, clinical manifestations begin in childhood and include acroparesthesias, telangiectasias, angiokeratomas, gastrointestinal symptoms, cornea verticillata, renal manifestations, heat and cold sensitivity, exercise intolerance, and hypohidrosis [5,6,7]. Progressive cardiac and cerebrovascular disease accounts for the majority of deaths [8].

Cerebrovascular involvement in FD may lead to transient ischemic attacks (TIA) and ischemic strokes, which occur in approximately 25% of patients, with a mean age at onset of 40 years [9]. Tortuosity and dilatation of large intracranial arteries (dolichoectasia) are also common [10], mainly affecting the basilar artery (BA) [11]. There is also evidence of a resting cerebrovascular hyperperfusion in FD males, which may cause increased shear stress and vessel wall damage over time [12]. Finally, severe, progressive, and confluent white matter lesions (WMLs) are the most prominent neuroimaging findings in FD, with posterior circulation being more prone to vascular lesions and perfusion deficit [13]. WMLs can occur from early age [14] and are usually asymptomatic or associated with subtle neuropsychological deficits [15].

The pathogenesis of WMLs in FD is still debated. The most convincing hypothesis states that increased cerebral blood flow (CBF) and altered vascular reactivity due to Gb3 deposition may lead to gliosis, demyelination, and increased interstitial water content, eventually resulting in WMLs [16]. However, the pathophysiological model accounting for the development of WMLs also requires the condition of chronic cerebral hypoperfusion [17].

Enzyme replacement therapy (ERT) improves renal and cardiac function in FD, whereas the beneficial effects on WMLs or preventing cerebrovascular events have not yet have been demonstrated [11]. A reasonable explanation accounting for this lack of efficacy may lie in the restricted access of the infused enzyme to the vascular system through the endothelial cells of the brain–blood barrier (BBB) [18]. Regarding chaperon therapy, it is currently unclear whether migalastat might be effective in reducing the risk of stroke in FD patients [19]. However, since migalastat is able to cross the BBB, it might contribute to reduce the occurrence of cerebrovascular events and WML load [20]. Nevertheless, in the phase III ATTRACT study, the low proportion of patients with cerebrovascular events (one in the ERT group and none with migalastat) does not allow any definite conclusions to be drawn [21]. Therefore, as recently suggested by practical recommendations [22], treatment with migalastat can be considered in patients with FD aged ≥16 years with amenable mutations and with TIAs/strokes and/or WMLs, although further studies including larger populations are necessary.

### 1.2. Transcranial Doppler Sonography

Hemodynamically, FD shows a prominent thickening of the common carotid artery intima, an impairment of the cerebrovascular autoregulation and vasoreactivity, and a cerebral hyperperfusion in the posterior circulation, all of which seem to play a significant role in the pathophysiology of neurovascular manifestations of the disease [23].

Transcranial Doppler sonography (TCD) is a noninvasive ultrasound technique that uses a low-frequency (≤ 2 MHz) transducer probe to insonate the basal cerebral arteries through relatively thin bone windows. TCD allows in vivo monitoring of CBF velocity and vessel resistance over an extended time and with a high temporal resolution. Most supporting evidence concerns the prognostication and initiation of preventive strategies in sickle cell disease [24], subarachnoid hemorrhage [25], stroke [26], thrombolysis in brain ischemia [27], intensive care neuromonitoring [28], diagnosis of brainstem death, and right-to-left cardiopulmonary shunt [29]. More recently, we have used TCD to provide useful indices of occurrence and severity of small vessel disease and executive dysfunction in elderly patients at risk for future dementia [30,31]. 

TCD is also used to investigate the cerebral pressure autoregulation [32]: combined with waveform morphology, some indices derived from the flow velocities, such as the Gosling’s pulsatility index (PI) and the Pourcelot resistivity index (RI), allow for identification of increased cerebrovascular resistance, vasospasm, and hyperdynamic flow states. 

Cerebral vasoreactivity (CVR) can be accurately and noninvasively investigated by TCD through assessment of the vasodilatory or vasoconstrictive capacity of the resistance arterioles of the brain [33,34,35]. The modulation of systemic blood pressure (and, therewith, the cerebral perfusion pressure) is suitable for investigating the cerebral autoregulatory response, whereas other stimuli (such as acetazolamide administration and CO_2_ inhalation) evoke a hemodynamic response that is related to the metabolic reaction of the brain due to the stimulus itself [34,36,37]. In recent years, more physiological stimuli for assessing cerebral vasoreactivity have been used, such as breath-holding and voluntary hyperventilation [38], which are well tolerated and recommended for CVR testing in patients with stable pulmonary conditions and diseases affecting cerebral microvasculature [35,39].

### 1.3. Aim and Hypothesis

Although the neurovascular burden is often clinically relevant, both at disease onset and during its course, negatively affecting health and quality of life [15], only few studies to date have addressed whether asymptomatic FD (aFD) subjects already have microcirculation changes [40]. In this scenario, the detection of minimal or subclinical changes in cerebral hemodynamics may allow an early diagnosis and the initiation of preventive and therapeutic strategies that might modify the natural history of the cerebrovascular disease in FD. Moreover, conflicting findings regarding CBF velocity changes in FD by TCD have been published. A decreased CBF velocity has been reported by Hilz et al. [41] and Azevedo et al. [40] in symptomatic and presymptomatic subjects, respectively. On the other hand, Moore et al. [42] found increased velocities in FD that reversed after ERT. Conversely, normal CBF velocity was reported by Segura et al. [43] and Uçeyler et al. [44], who did not observe changes after ERT [44]. 

In this study, we assessed the TCD pattern in aFD subjects compared to healthy controls. We hypothesized that TCD might be able to identify preclinical CBF changes, thus providing neurosonological evidence of “brain at risk” of cerebrovascular complications in the course of FD.

## 2. Materials and Methods

### 2.1. Subjects and Assessment

A sample of 60 participants (age > 18 years) was consecutively recruited by the Neurosonology Laboratory of the “Azienda Ospedaliera Universitaria Policlinico Gaspare Rodolico-San Marco” of Catania (Italy) and referred by the “Multidisciplinary Center for the Diagnosis and Treatment of Fabry’s disease” of the University of Catania. 

From the original sample of 32 aFD subjects, two were excluded due to bilateral absence of an adequate transtemporal window for TCD examination. Therefore, the aFD group included 30 subjects with a genetic diagnosis of FD (19 females, mean age ± standard deviation: 37.97 ± 11.95 years), who were identified among the family members of the symptomatic probands. The mutations, all related to FD with cerebrovascular involvement [45,46,47,48,49,50], were as follows: D313Y in 6 patients; F113L in 5, A143T in 11, S126G in 4, M51I in 2, and G395A in 2. The determination of LysoGb3 in blood was performed as previously described by Polo et al. [51]. Briefly, LysoGb3 levels were measured by reversed-phase liquid chromatography. Mass spectrometry was carried out with the detector set in positive mode using an electrospray ionization source.

Exclusion criteria were presence of neurological signs or symptoms; evidence of WMLs or any other neuroradiological lesion from brain magnetic resonance imaging (MRI); bilateral insufficient acoustic bone windows; any neurological or psychiatric disease; any severe or not compensated medical illness; alcohol or drug abuse. The control group included 28 age-and-sex-matched healthy volunteers (16 females, mean age ± standard deviation: 39.0 ± 10.31 years). All participants were screened for cardio- and cerebrovascular risk factors through clinical records, had normal Doppler ultrasound images of the extracranial vessels, and were drug-free. 

Brain MRI was performed in all subjects using a 1.5-T General Electric system. Angiography scan was carried out by using the non-contrast 3D time-of-flight method with maximum intensity projection sequences. The slice thickness was 5 mm with a 0.5 mm slice gap. 

All participants gave their signed informed consent prior to the inclusion in the study, which was approved by the Ethics Committee of the “Azienda Ospedaliera Universitaria Policlinico Gaspare Rodolico-San Marco” (approval code: n. 9/2018/PO) and carried out in accordance with the Declaration of Helsinki of 1964 and later amendments.

### 2.2. TCD Procedure

TCD was performed by a trained sonographer (RB) in all participants, with the same system and under the same experimental conditions. The operator was blind with respect to the group allocation of participants. CBF velocity of BA and proximal tract (M1) of the middle cerebral artery (MCA) was recorded with a handheld 2 MHz DWL ultrasound probe pulsed-wave Doppler through the suboccipital and transtemporal bone window, respectively, at rest and at the depth that provided the best signal (50–60 mm for the MCA and 80–90 mm for the BA). The following parameters were evaluated: peak systolic blood flow velocity (PSV), end-diastolic blood flow velocity (EDV), mean blood flow velocity (MBFV), PI (according to the formula (PSV − EDV)/MBFV) [52], and RI (that was equal to (PSV − EDV)/PSV). TCD values were acquired after a 30 s stable recording period lasting for at least 10 cardiac cycles [53]. 

CVR by the 30 s breath-holding test was also evaluated for the MCA, bilaterally. The vasodilatory stimulation via the breath-holding and the CO_2_-induced hypercapnia can detect impaired cerebral vasomotor reserve, and an altered response may outline an increased risk of stroke [54]. Subjects were instructed to hold their breath for 30 s after a normal inspiration without doing a Valsalva maneuver; then, the breath-holding index was calculated as follows: [(PSV after breath holding − PSV at rest)/PSV at rest/time of breath holding] × 100. This procedure was repeated after a resting period of two minutes, and the mean of the two values was taken for analysis [55]. The MCA-derived parameters, both at rest and during the CVR task, were obtained as the mean of two measurements on each side. 

All data were collected and stored on a dedicated PC for offline analysis.

### 2.3. Statistical Analysis

For the statistical analysis, between-group comparisons were performed by means of the Student’s *t*-test after assessing the normality of data distribution (Kolmogorov–Smirnov and Lilliefors tests for normality). A post hoc power analysis was also carried out showing that with the available sample sizes, we were able to detect a significant difference with alpha 0.05 and power 80% for comparisons characterized by an effect size of 0.75. Differences were considered significant when they were below the level of *p* < 0.05. Correlations were analyzed by calculating the Pearson correlation coefficient and differences in frequencies by means of Fisher’s exact test.

## 3. Results

Demographic features were similar between aFD subjects and controls (Table 1). One aFD subject and one control had mild dyslipidemia, whereas one aFD subject and two controls were former smokers; none of the participants had risk factors other than those reported, as confirmed by clinical records and recent routine laboratory tests. Heart rate, mean arterial pressure, and oxygen saturation by pulse oximetry device, recorded at the time of the examination, and were normal in all cases. Brain MRI was normal in all participants, and none had ectasia, elongation, or tortuosity of BA. 

As shown in Table 2, no difference between groups was observed for any TCD measure from MCA, both at rest and after the breath-holding test. A significantly higher MBFV (*p* = 0.035) and a reduced RI (*p* = 0.0002) from BA were found in aFD subjects compared to controls. PI from BA was reduced in aFD subjects with respect to controls, although without reaching a statistical significance (*p* = 0.053). No correlation was observed between any BA-derived parameter and LysoGb3 values.

## 4. Discussion

### 4.1. Main Findings

The main finding of this study is an increase in MBFV and a reduction of RI from BA in aFD subjects compared to healthy controls. As known, MBFV is a relevant TCD measure which can be affected by a number of physiological and pathophysiological factors. An increased value is usually observed in vessel stenosis, vasospasm, or hyperdynamic flow, whereas a decrease may indicate hypotension, reduced CBF, intracranial pressure, or brainstem death. PI and RI are also influenced by some physiological factors, such as arterial pressure, vascular compliance, and changes in CO_2_ partial pressure. Indeed, PI, normally ranging between 0.5 and 1.19, provides information on downstream vascular resistance [52,56]. Proximal stenosis or occlusion may at lower PI below 0.5, due to downstream arteriolar vasodilation, while distal occlusion or constriction may increase PI above 1.19 [57]. Changes in RI reflect a disease pattern similar to that observed in case of abnormal PI. Namely, RI values > 0.8 indicate increased downstream resistance, while hyperemia, arteriovenous malformation, vasospasm, and rewarming following hypothermia can lead to decreased RI [58,59]. Vasodilation stimulation via breath-holding and CO_2_-induced hypercapnia can detect impaired cerebral vasomotor reserve and predict the risk of stroke [54].

Previous studies evaluating TCD in FD reported heterogeneous findings. Hilz et al. [41], investigating the MCA of 22 asymptomatic young males, found a reduced MBFV. Moore et al. [60] examined 63 hemizygous FD males compared with 31 male controls and found elevated CBF velocities in M1 and in the posterior cerebral artery (PCA), although no data on vascular risk factors and brain MRI were reported. Uçeyler et al. [44] analyzing all TCD parameters (PSV, EDV, MBFV, RI, and PI) from MCA, PCA, anterior cerebral artery (ACA), and BA of 68 FD patients and 77 controls, found no significant difference between groups. However, 23 out of 68 patients were on ERT, and no data on brain MRI were available. Segura et al. [43] found no significant changes of cerebral hemodynamics in 10 FD patients with a classic phenotype and 17 healthy volunteers, although RI from BA was not considered in their analysis. Two previous studies assessed the effect of breath-holding on MCA vasoreactivity in symptomatic FD patients [42,43]. Moore et al. [42] found no significant difference in TCD parameters from M1 before and after a breath-holding of one minute, whereas Segura et al. [44] observed a non-significant trend towards a lower vasomotor reactivity in FD compared to controls. In line with these studies, we did not find any difference between groups for MCA parameters of CBF velocity and CVR. Therefore, given that even patients with a classic phenotype exhibited normal CVR at the breath-holding test, it is reasonable to hypothesize a similar result also in asymptomatic subjects. Overall, previous TCD data in FD are conflicting as the examined groups are not homogeneous in terms of clinical features and TCD methodology, and neuroimaging correlates are often lacking.

Our results suggest that an altered CBF velocity in posterior circulation may occur even in aFD subjects, and that TCD is able to detect early CBF changes. The low RI and PI might be interpreted as increased CBF volume accompanied by increased flow velocity, as also observed in other disease models [61]. It is also well known that FD patients have a greater susceptibility to the involvement of the posterior cerebral circulation. Vertebral–basilar arterial dolichoectasia is common in neuroimaging studies (38% in males and 57% in females according to Rolfs et al.; up to 87% in the study by Fellgiebel et al. [62,63]) and can occur relatively early in the disease course. Moreover, Fellgiebel et al. [64] suggested an enlarged BA diameter in FD patients with stroke as a sensitive screening tool of FD. Recently, a larger BA diameter has been associated with a higher risk of stroke in FD [65]. Lastly, the annual BA diameter change seems to be inversely correlated with the duration of ERT in males [66].

Dysfunctional cerebral circulation in FD has been demonstrated by several studies using imaging end-points, such as CBF, CBF velocity, CVR, and functional MRI [12,16,42,67,68,69]. These studies found significant cerebral hyperperfusion in FD, predominantly in the posterior circulation. Interestingly, hyperperfusion is not observed in systemic circulation, as demonstrated by normal cardiac output in these patients [12]. Notably, Moore et al. [16] subsequently demonstrated that cerebral hyperperfusion was a vascular phenomenon, not caused by neuronal overactivity. 

Taken together, we can conclude that neurovascular involvement in FD may not be associated with major cerebrovascular events only, but also with microstructural and functional changes, as shown by widespread changes in the default mode network at functional MRI [69]. The present study further supports the presence of an altered CBF velocity in the posterior circulation of aFD subjects at risk for cerebrovascular complications.

### 4.2. Strengths and Limitations

A strength of this study is the inclusion of a relatively large sample of aFD subjects, which is a rare genetic disorder. 

A limitation is that aFD subjects belonged to six families only and, therefore, it remains to be determined to what extent these results can be generalized to all aFD subjects.

A second limitation is that quantitative analysis of the MRI angiograms is lacking, although any vertebral–basilar dolichoectasia was excluded in all patients by a trained neuroradiologist.

Another caveat is that CVR of BA was not assessed. Most investigations focused on CVR of MCA, as TCD provides easy and reliable sampling of flow velocities in this artery [33]; conversely, few studies only have investigated CVR of BA and reference values are not standardized [70]. Moreover, there are conflicting results on the reactivity in BA, since a previous TCD study in normal individuals found a reduced BA reactivity in response to step hypoxia compared to that of MCA [71], whereas a subsequent study demonstrated no difference between CVR of BA and MCA during breath-holding [70]. Nevertheless, given the finding of cerebrovascular dysfunction in the posterior circulation of FD, the assessment of CVR of BA and the comparison with that of MCA would perhaps have disclosed additional results in this specific group of patients.

Finally, TCD parameters were not measured from ACA and PCA; however, MCA and BA perfuse the largest anterior and posterior territories of the brain, respectively, and are ideally located for TCD recording, thus providing reliable and satisfactory velocities and vessel resistance.

## 5. Conclusions

FD is a multifaceted inherited disease, of which stroke may be the first manifestation and a significant cause of death. The early detection of cerebrovascular involvement can potentially improve the management of still asymptomatic subjects; thus, it is reasonable to expect a potential prevention of future complications and disability. TCD screening is a useful additional tool to noninvasively probe and monitor the cerebrovascular correlates of FD. Further studies in larger family pedigrees are needed to confirm and extend the early impairment of cerebral hemodynamics and its impact on therapeutic interventions.

## Figures and Tables

**Table 1:** Clinical and demographic features of participants.

Variable	aFD (*n* = 30)	Controls (*n* = 28)	Fisher’s Exact Test
	*n* (%)	*n* (%)	*p*
Age, Years *	37.97 ± 11.95	39.0 ± 10.31	NS
Sex, Females	19 (63.3%)	16 (57.1%)	NS
Dyslipidemia	2 (6.6%)	1 (3.6%)	NS
Former Smokers	1 (3.3%)	2 (7.1%)	NS

*Legend:* aFD = asymptomatic Fabry’s disease subjects; NS = not significant; * mean ± SD and Student’s *t*-test.

**Table 2:** TCD data of both groups.

TCD Index	aFD		Controls		Student’s *t*-Test	
	Mean	SD	Mean	SD	*t* Value	*p*
MCA PSV	93.27	19.08	92.45	18.34	0.167	0.868
MCA MBFV	62.55	13.15	61.39	10.79	0.363	0.718
MCA EDV	42.80	9.72	42.95	11.58	−0.054	0.957
MCA PI	0.81	0.09	0.80	0.07	0.791	0.432
MCA RI	0.55	0.04	0.55	0.04	0.018	0.985
aMCA PSV	102.42	20.38	100.45	18.15	0.388	0.700
aMCA MBFV	70.36	16.13	69.70	13.18	0.171	0.865
aMCA EDV	49.60	12.08	50.14	12.72	−0.168	0.867
aMCA PI	0.75	0.10	0.76	0.10	−0.135	0.893
aMCA RI	0.53	0.06	0.53	0.06	−0.020	0.984
MCA BHI	0.43	0.52	0.46	0.38	−0.179	0.859
BA PSV	66.40	15.99	58.93	13.63	1.909	0.061
BA MBFV	44.55	11.31	38.75	8.93	2.157	**0.035**
BA EDV	30.20	8.54	25.61	9.86	1.901	0.062
BA PI	0.79	0.13	0.86	0.15	−1.979	*0.053*
BA RI	0.53	0.06	0.61	0.08	−3.978	**0.0002**

*Legend (in alphabetic order)*: a = after breath-holding; aFD = asymptomatic Fabry’s disease subjects; BHI = breath-holding index; MCA = middle cerebral artery; BA = basilar artery; PSV = peak systolic blood flow velocity; EDV = end-diastolic blood flow velocity; MBFV = mean blood flow velocity; PI = pulsatility index; RI = resistivity index; SD = standard deviation; TCD = transcranial Doppler sonography; numbers in bold = statistically significant *p* values; numbers in italics: trend towards a significant *p* value.
